# Effectiveness of the WHO-Authorized COVID-19 Vaccines: A Rapid Review of Global Reports till 30 June 2021

**DOI:** 10.3390/vaccines9121489

**Published:** 2021-12-16

**Authors:** Chang-Jie Cheng, Chun-Yi Lu, Ya-Hui Chang, Yu Sun, Hai-Jui Chu, Chun-Yu Lee, Chang-Hsiu Liu, Cheng-Huai Lin, Chien-Jung Lu, Chung-Yi Li

**Affiliations:** 1 Department of Neurology, En Chu Kong Hospital, New Taipei City 237, Taiwan; 117223@ntuh.gov.tw (C.-J.C.); 01480@km.eck.org.tw (H.-J.C.); 01548@km.eck.org.tw (C.-Y.L.); 00520@km.eck.org.tw (C.-H.L.); 00202@km.eck.org.tw (C.-H.L.); 01181@km.eck.org.tw (C.-J.L.); 2 Department of Neurology, National Taiwan University Hospital, Taipei 100, Taiwan; 3 Division of Pediatric Infectious Diseases, Department of Pediatrics, National Taiwan University Hospital, Taipei 100, Taiwan; cylu@ntu.edu.tw; 4 College of Medicine, National Taiwan University, Taipei 100, Taiwan; 5 Department of Public Health, College of Medicine, National Cheng Kung University, Tainan 701, Taiwan; t88071016@gs.ncku.edu.tw; 6 Department of Public Health, College of Public Health, China Medical University, Taichung 404, Taiwan; 7 Department of Healthcare Administration, College of Medical and Health Science, Asia University, Taichung 413, Taiwan

**Keywords:** COVID-19, COVID-19 vaccines, SARS-CoV-2 variants

## Abstract

Large clinical trials have proven the efficacy of the COVID-19 vaccine, and the number of studies about the effectiveness rapidly grew in the first half of the year after mass vaccination was administrated globally. This rapid review aims to provide evidence syntheses as a means to complement the current evidence on the vaccine effectiveness (VE) against various outcomes in real-world settings. Databases (PubMed, EMBASE, and MedRxiv) were searched up to 30 June 2021, (PROSPERO ID: 266866). A total of 39 studies were included, covering over 15 million participants from 11 nations. Among the general population being fully vaccinated, the VE against symptomatic SARS-CoV-2 infection was estimated at 89–97%, 92% (95% CI, 78–97%), and 94% (95% CI, 86–97%) for BNT162b2, ChAdOx1, and mRNA-1273, respectively. As for the protective effects against B.1.617.2-related symptomatic infection, the VE was 88% (95% CI, 85.3–90.1%) by BNT162b2 and 67.0% (95% CI, 61.3–71.8%) by ChAdOx1 after full vaccination. This review revealed a consistently high effectiveness of certain vaccines among the general population in real-world settings. However, scarce data on the major variants of SARS-CoV-2 and the shortness of the study time may limit the conclusions to the mRNA vaccines and ChAdOx1.

## 1. Introduction

Large randomized control trials have demonstrated the efficacy against SARS-CoV-2 infection to be 70%, 94%, 95%, 78%, and 84% after two doses of the ChAdOx1 (Oxford/AstraZeneca, Oxford, UK), mRNA-1273 (Moderna, Cambridge, MA, USA), BNT162b2 (BioNTech/Pfizer, Mainz, Germany), BBIBP-CorV (Sinopharm, Beijing, China), and CoronaVac (Sinovac, Beijing, China) vaccines, respectively [1,2,3,4,5]. A single dose of the Ad26.COV2.S (Johnson & Johnson, New Brunswick, NJ, USA) vaccine yielded an efficacy of 82% against severe-critical COVID-19 disease [6]. Based on the good results from clinical trials, COVID-19 vaccination programs have been extensively rolled out in many countries around the world. However, over the first half-year since vaccine administration globally, most countries have full vaccination rates of less than 50% [7]. Billions of people in the world are eagerly waiting for the COVID-19 vaccines on the one hand and questioning how well the vaccines work in the real world on the other hand. As relevant study reports have been released successively, a wide range of vaccine effectiveness (VE) has been noticed. The estimated effectiveness after full vaccination could range from 50% [8] to 100% [9], according to effectiveness studies of the six vaccines (BNT162b2, ChAdOx1, Ad26.COV2.S, mRNA-1273, BBIBP-CorV, CoronaVac), which were listed on the World Health Organization (WHO) Emergency Use Listing as of June 2021 [10].

Given a different efficacy was shown for vaccines developed with different platforms [1,2,3,4], their effectiveness in the real world for different populations needs to be confirmed. A Denmark study showed different VE in different age groups (77% for people ≥ 85 years of age vs. 86% for people ≥ 65 years), and different living/working environments (53% for long-term care facilities dwellers vs. 80% for healthcare workers) [11]. Similarly, the VE may also be different among different countries or races. Attention should also be paid when VE from different studies are compared as VE of a given vaccine depends on what outcomes were chosen in determining VE. A study on the residents of long-term care facilities in Spain showed the VE of mRNA vaccines was 70% against asymptomatic infection and 97% against death [12]. In addition, many SARS-CoV-2 variants have been evolving since the pandemic. B.1.1.7 (alpha), B.1.351 (beta), B.1.617.2 (delta), and P.1 (gamma) variants, which are circulating in the whole world and are causing serious infections and mortality rate that are classified as variants of concern (VOC) by WHO. The VE against these VOC needs to be explored from the literature.

This rapid review aimed to assess the effectiveness of WHO-authorized COVID-19 vaccines, taking into account the aforementioned factors including country, characteristics of the study population, study design, outcomes, and the analysis of the involved VOC.

## 2. Materials and Methods

This rapid review was conducted based on the updated guideline of PRISMA 2020 statement and its recommended checklist [13,14]. We registered this review on PROSPERO (ID: 266866) on 13 July 2021. The eligibility criteria are reports evaluating the effectiveness of COVID-19 vaccines in populations aged ≥16 years. Additional inclusion criteria require that the sample size of the vaccinated population should be more than 1000 to have a sufficient event number. Two authors (C.-J.C., Y.S.) performed the literature search in PubMed, EMBASE, and medRxiv. The search terms in PubMed and EMBASE were “effectiveness”, “COVID-19 vaccine”, and publish time “2021”. Preprint articles from medRxiv were searched with the terms “effectiveness COVID-19 vaccine” or “effectiveness SARS-CoV-2 vaccine” in the titles or abstracts.

The article types we reviewed included original investigations, research letters, short communications, and correspondence articles. While screening the title and abstracts of the relevant articles in PubMed, similar research with titles shown on the web page was also checked. We updated our search up to 30 June 2021. C.J.C. and Y.S. contributed to the title and abstract screening for relevance and reviewing of full-text articles against inclusion and exclusion criteria. We excluded the following research: in vitro studies, animal studies, experimental clinical trials, systematic reviews or meta-analyses, diagnostic studies, methodological publications, editorial-style reviews, abstracts of posters, secondary analyses, studies with only immunogenicity data, safety reports or post-infection treatment, and articles with analyses only on a very specific target population, such as veterans, dentists, pregnant women, and patients with malignancy or mental illness.

Ethical approval was not applicable because all the study materials of this review are from the published articles and preprints.

After identification of all relevant articles, quality assessment was performed based on ROBINS-I of Cochrane Handbook to assess the risk of bias. Each bias domain and overall risk of bias was judged as “Low”, “Moderate”, “Serious”, or “Critical” risk of bias based on the check list on the ROBINS-I assessment chart. The extracted data included the following items: author, country, number of vaccinated and unvaccinated participants, study design, age and characteristic of participants, types of vaccine, outcomes, definition about minimal intervals between vaccination (first dose and second dose), and event measurement, involvement of SARS-CoV-2 VOC, and VE with confidence interval (CI) respectively after first dose and second dose of vaccine.

The formula for calculating VE is (1 − hazard ratio for SARS-CoV-2 infection in vaccinated vs. unvaccinated participants) × 100%. In studies that reported the incidence of infection, we calculated the incidence rate ratio (IRR) and converted it to unadjusted VE as (1 − IRR in vaccinated vs. unvaccinated participants) × 100%. The VE from a case-control study was calculated as (1 − odds ratio) × 100%. In case of insufficient data in an article, we contacted the authors to obtain the required information by email. We applied narrative synthesis to process the data from the included studies. As the number of studies of VE constantly grew over our processing period, this review only included reports that were released before 1 July 2021. Distributions of the VE estimates derived from the included studies were further graphically presented by Box plots, according to the study population, brand of vaccine, variant, number of doses, and outcome.

## 3. Results

### 3.1. Study Selection and Characteristics

**Figure 1:**
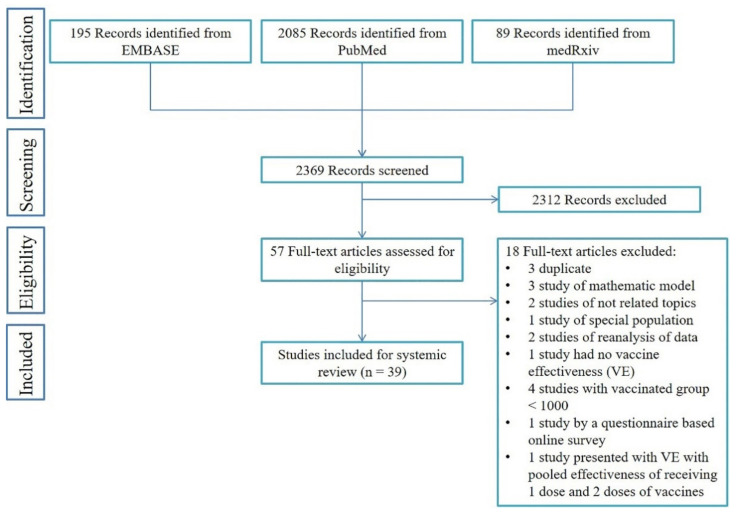
PRISMA flowchart of the literature searches.

Of 2369 searched articles (2085 from PubMed, 195 from EMBASE, and 89 preprints from MedRxiv), 2312 were excluded while screening the abstracts and titles. After a full-text review of the remaining 57 articles for eligibility, 18 were removed due to not completely meeting the criteria. Therefore, 39 studies [9,11,12,15,16,17,18,19,20,21,22,23,24,25,26,27,28,29,30,31,32,33,34,35,36,37,38,39,40,41,42,43,44,45,46,47,48,49,50] that met the inclusion criteria were included in this rapid review (Figure 1, Supplementary Table S1). The results of the quality assessment of all the included studies are shown in Supplementary Table S2. Before 1 July 2021, 24 of 39 studies were published in peer-reviewed journals [9,12,15,16,17,19,22,24,25,28,29,30,31,33,34,36,37,40,41,42,45,47,48,50] while the remaining 15 studies were posted online as preprint articles [11,18,20,21,23,26,27,32,35,38,39,43,44,46,49]. The characteristics of the studies are shown in Supplementary Table S1, including the country, study design, types of vaccines, outcomes, and SARS-CoV-2 variants involved in the studies. The outcomes include all laboratory-confirmed SARS-CoV-2 infection, asymptomatic and symptomatic infection, hospitalization, critical disease, and death. As for evaluating the protective effects of the vaccine on SARS-CoV-2 variants, 5 studies mentioned the approximate prevalence of variants in the region of the study population [18,19,28,34,49], and 11 studies calculated the number or percentage of cases with variants among all or sampled participants with a positive test of SARS-CoV-2 variants [20,26,31,33,36,37,38,42,43,46,48]. Only three studies evaluated the VE against specific VOC, in which one study from Qatar reported the VE against B.1.1.7 and B.1.351 [9], one study from Canada studied VE against B.1.1.7 and P.1 [46], and the other one study from the UK evaluated the VE against B.1.1.7 and B.1.617.2 (delta variant) [20].

### 3.2. Effectiveness after Partial or Full Vaccination for Various Outcome of Interest

#### 3.2.1. Overall SARS-CoV-2 Infection across General and Sub-Populations

**Figure 2:**
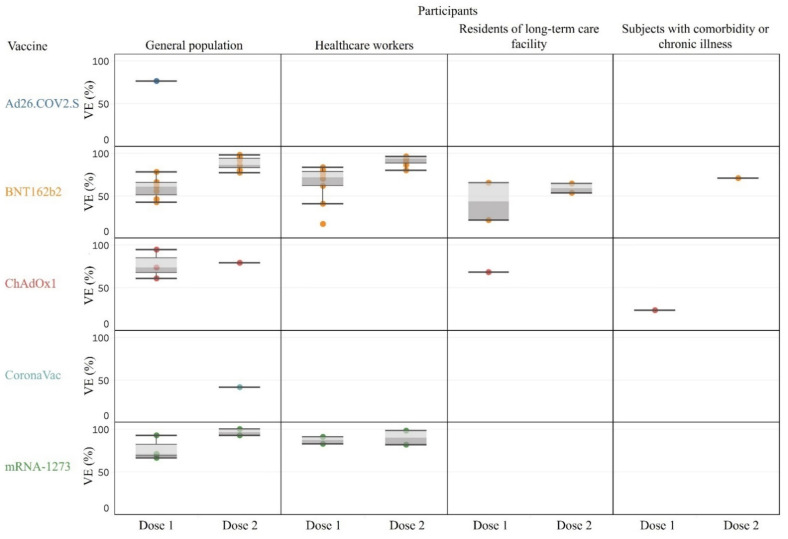
Vaccine effectiveness against overall SARS-CoV-2 infection.

The characteristics of the studies and their VE estimates with 95% CI against overall SARS-CoV-2 infection among general and specific populations are shown in Table 1 and the distributions of the VE estimates are shown in Figure 2. Among the partially vaccinated general population, the VE varied among studies (42–78% by BNT162b2 [23,28,31,32,40,42,46], 67–93% by mRNA-1273 [26,31,40], and 61–95% by ChAdOx1 [31,32,42]). After full vaccination, the VE was all higher than 75% (77–98% by BNT162b2 [11,23,28,31,33,40,42], 93–99% by mRNA-1273 [31,40], 79% by ChAdOx1 [42], and 78% by Ad26.COV2.S with a single dose as full vaccination [27]). The only exception was a low VE noted for CoronaVac (42%, 95% CI, 26.9–53.3%) in a Brazilian study of subjects aged ≥70, with an 83% P.1 variant prevalence during the study time (Table 1) [43]. The VE among fully vaccinated healthcare workers was 80% or higher (80–97% by BNT162b2 [11,19,22,30,34,39,47,48] and 82–99% by mRNA-1273 [47,48]) (Table 1). Comparatively, VE estimates among residents of long-term care facilities were low (partially vaccinated: 21–65% by BNT162b2 [39,45], 68% [95% CI, 34–85%] by ChAdOx1 [45]; fully vaccinated: 53–64% by BNT162b2 [11,39]). Additionally, the VE was also relatively low among subjects with comorbidity or chronic illness (one dose of ChAdOx1: 24% after ≥21 days, 50% after ≥42 days [18], two doses of BNT162b2: 71% after ≥7 days [11]) (Table 1).

#### 3.2.2. Asymptomatic Infection, Symptomatic Infection, Hospitalization, Critical Disease, and Death

**Figure 3:**
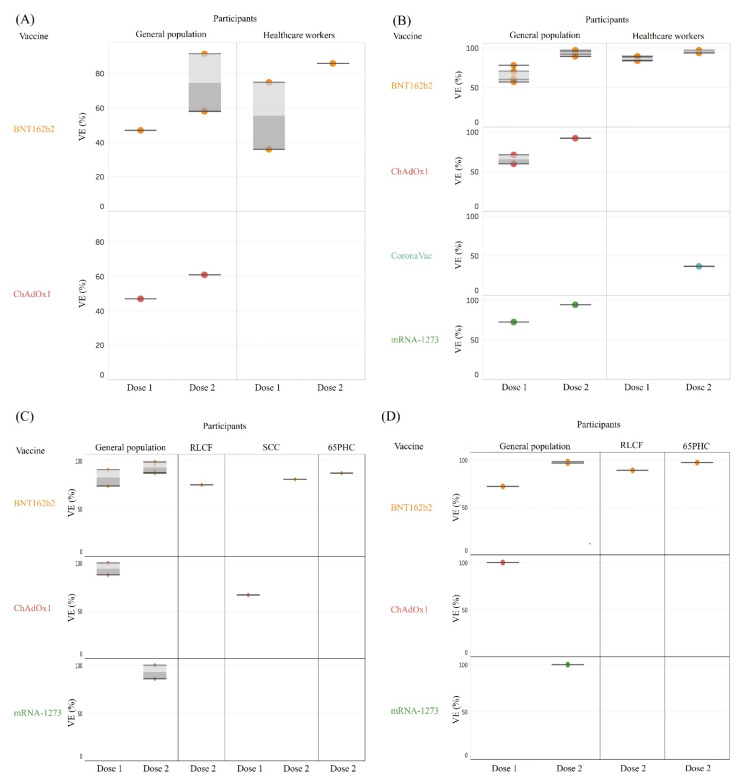
Vaccine effectiveness against various outcomes. (**A**) Vaccine effectiveness against asymptomatic infection. (**B**) Vaccine effectiveness against symptomatic infection. (**C**) Vaccine effectiveness against hospitalization. (**D**) Vaccine effectiveness against death. Abbreviations: RLCF: residents of long-term care facilities, SCC: subjects with comorbidity or chronic illness, 65PHC: individuals 65 years and older living at home but requiring practical help and personal care.

The VE estimates against SARS-CoV-2-related various outcomes are shown in Table 1, Figure 3A–D, and Supplementary Figure S1. Among the general population in the UK, the VE against asymptomatic infection after full vaccination either with BNT162b2 (VE: 58%) or ChAdOx1 (VE: 61%) was not as high as that in Israel (VE: 92%) (Figure 3A, Table 1A) [33,42]. Much more beneficial effects were found when symptomatic infection reduction was considered as an outcome, with the VE reaching up to 89–97% for BNT162b2 [26,28,33,37,42], 92% for ChAdOx1 [42], and 94% for mRNA-1273 after full vaccination (Table 1, Figure 3B) [26]. Studies in healthcare workers also showed beneficial effects from vaccination (VE: 94–97% by two doses of BNT162b2) [15,16,30], except for a CoronaVac study in Brazil, which showed a low VE against symptomatic infection with the predominant P.1 variant (37%, 95% CI, 54.9–74.2%) even after being fully vaccinated (Figure 3B, Table 1) [35]. The protective effects become more obvious for more severe outcomes. The VE against critical disease was higher than 90% by BNT162b2 [28,33,40]. A study even demonstrated a VE of 100% for subjects who were fully vaccinated with mRNA-1273 (see Supplementary Figure S1) [40].

Regarding the risk reduction on hospitalization among the general population, the effectiveness was similar among one dose of ChAdOx1 (VE: 88–100%) [31,50], two doses of BNT162b2 (VE: 87–99%) [11,28,33,50], and two doses of mRNA-1273 (VE: 86–100%) (Figure 3C) [31,40]. Reduction of death for more than 95% was also found by one dose of ChAdOx1 and two doses of mRNA vaccines (Figure 3D). The VE estimates against COVID-19 hospital admissions after vaccination either with BNT162b2 or ChAdOx1 in older age groups were slightly lower than that of younger age groups [50]. The result of VE across age groups is shown in Supplementary Table S3.

### 3.3. Effectiveness of Vaccines against SARS-CoV-2 VOC

**Figure 4:**
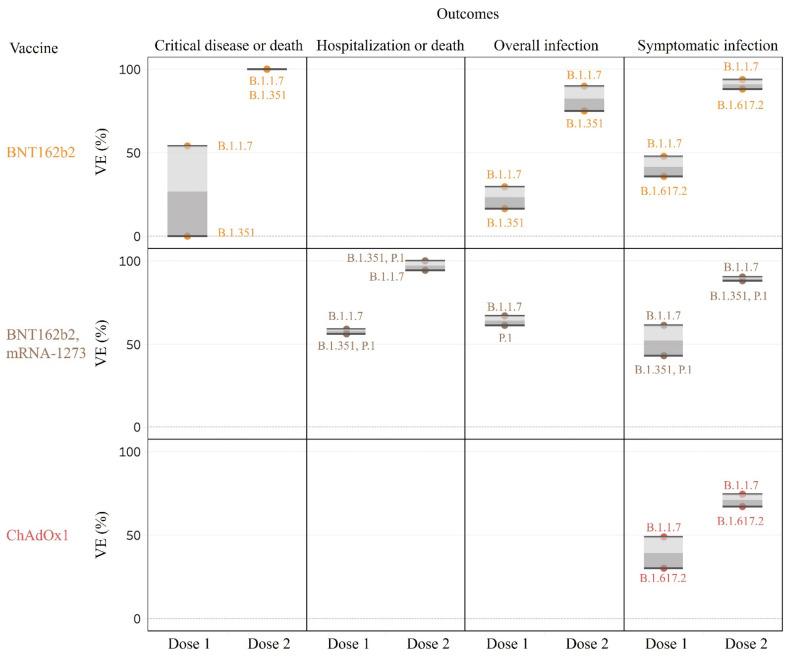
Vaccine effectiveness against SARS-CoV-2 variants of concern.

Three studies specifically calculated the VE against the VOC [9,20,46] (Table 1, Figure 4). For the Alpha variant (B.1.1.7), one dose of BNT162b2 or ChAdOx1 provided around 50% of VE on symptomatic infection and critical disease or death [9,32]. For the delta variant (B.1.617.2), better VE was also found for two doses of BNT162b2 (VE: 93.4%, 95% CI, 90.4–95.5%) than in ChAdOx1 (66.1%, 95% CI, 54.0–75.0%) in terms of preventing symptomatic infection [32].

## 4. Discussion

This rapid review included 39 studies from 11 countries. Some of these studies may have overlapping populations with data obtained from the same datasets (e.g., national registry) or from the same institution or same region [15,16,17,19,20,21,28,32,34,36,37,38,39,41,42,45]. By excluding possible double counted participants, this review approximately covered a population of 15 million in total with over 8 million vaccinated subjects. The results of all included studies consistently show that vaccination among the general population in real-world settings, with either BNT162b2, ChAdOx1, or mRNA-1273, substantially reduced the risk of overall SARS-CoV-2 infection. Though the effectiveness of the vaccine in residents of long-term care facilities is lower than that in the general population, studies showed that just one dose of the BNT162b2 or ChAdOx1 vaccine can still provide a VE of 60% or higher. Their protective effectiveness became more obvious as the severity of the clinical endpoint increased. The effectiveness of one dose of ChAdOx1 in preventing hospitalization and death was higher than 80% in the general population and similar to that observed in subjects fully vaccinated with BNT162b2 or mRNA-1273. While the delta variant is becoming the dominant strain circulating in many parts of the world, VE reports on the delta variant are scarce. One recently published study shows that two doses of BNT162b2 can significantly reduce the risk of delta variant-related symptomatic infections [20].

The suboptimal VE observed in the residents of long-term care facilities is probably due to immunosenescence, which leads to defects in innate and adaptive immune responses. Vaccine responses tended to be weaker and declined earlier [51] with a lower titer of antibodies [52,53,54]. Since they are vulnerable to developing SARS-CoV-2 infections and its complications, improved vaccine strategies or further vaccine boosting are required in these vulnerable populations.

It should be noted that VE between vaccines cannot be directly compared across these papers due to the following reasons. The first one is the heterogeneity in the interval between vaccination and the start of event measurement. The VE of the first dose of BNT162b2 against infection was 61% ≥14 days after the vaccination in the study by Pawlowski et al. [40] and 72% ≥21 days after vaccination in the study by Hall et al. [34]. As immunity is gradually building [3], the protective effects would increase over a given period [18,28]. At present, there is no data to suggest the timing of the VE plateau for each vaccine. Second, large variation was noted regarding the dosing intervals. To vaccinate as many people as possible, the UK government decided that the dosing interval for vaccines (whether BNT162b2 or ChAdOx1) could be extended up to 12 weeks [21,37]. In Canada, the administration of the second dose of BNT162b2 and mRNA-1273 was delayed by up to 16 weeks for most individuals due to the disruption of the vaccine supply [26]. There is limited data about the change of the effectiveness for extended dosing intervals. Variations in dosing intervals would be very common in most countries of the world due to the insufficient and unstable vaccine supply. Evidence regarding the effectiveness with appropriate and tolerable dosing intervals are needed [55,56]. Third, some studies [12,29,38,41,44,49] estimated the VE by pooled analyses of two vaccines, in which we cannot obtain the VE of each type of vaccine. Fourth, population bias with unmatched control arms or unequal use of vaccine types across study cohorts could make the data of direct comparison of vaccine types unreliable [20,32,37,40,50]. Different vaccines may target different populations in the roll-out period. For example, old age and healthcare workers are in the priority group of vaccination programs in most countries, but the timescale of supply was different between vaccine types [18,32]. Although the demographic factors were controlled in most studies, residual confounding may not be entirely excluded.

There are several barriers and limitations in this study. First, the literature about the VE against COVID-19 is rapidly evolving and growing exponentially. In this review, 15 of the 39 included studies were preprint articles up to 30 June 2021. While we were processing the review and extracting the data, in 5 July studies were accepted with their data changed in an online version [20,32,35,38,46,57] so that we need to constantly update the data accordingly before submission. Second, these included studies were disproportionately from countries in Europe, North America, and Israel, and were mostly focused on BNT162b2, because these countries had higher priority and access to a large amount of COVID-19 vaccines than most countries in other regions in the first half of 2021 [7]. In addition, shorter dosing intervals and a larger vaccine supply in these nations also make the data of VE associated with BNT162b2 much more comprehensive than other vaccines. Longer dosing intervals made the full vaccination reports of ChAdOx1 much fewer in number than those on mRNA vaccines among the included articles over the past months. Furthermore, the VE assessment reports on CoronaVac and Ad26.COV2.S are also too few to draw conclusions. Third, the VE estimates of all the studies were variable and mostly based on a relatively short study time (range, <2 months to 6 months) with a median follow-up period ranging from 28 to 106 days [15,16,22,33,34,39,40,47]. Further studies are needed to clarify the duration of the protective effects of vaccines. Fourth, studies focused on the VE against the variants of concern, in particularly the delta variant, which evolved as the dominant variant of SARS-CoV-2 in the first half-year of 2021, are scarce [32]. Additionally, the prevalence of the major variants is believed to be quite different among countries and subject to change over time, while the effectiveness of vaccines during the study time might be highly influenced by the local prevalence of the variants [43]. The heterogeneity across studies in the dosing interval, timing of outcome measurement, different target population between vaccine types, variations and shortness of study follow-up period, and scarce data about the prevalence of and the VE against major variants of SARS-CoV-2 all make meta-analyses for studies before 30 June 2021 extremely difficult and may possibly lead to biased results.

## 5. Conclusions

This rapid review provided timely and comprehensive evidence on the effectiveness of the ChAdOx1, BNT162b2, and mRNA-1273 vaccines against various COVID-19 infection-related endpoints ranging from asymptomatic to critical illness and death. This review highlights the VE across different segments of populations. These real-world results tend to support the implementation of mass vaccination campaigns as public health strategies, and may also help ease skepticism about VE, which is still a common problem in many parts of the world. However, data based on the studies of the first half-year of vaccine administration are not enough and may limit the conclusions regarding the mRNA vaccines and ChAdOx1. Further studies are needed to provide the information on different races/ethnicity, the effects against SARS-CoV-2 variants, and the duration of protection with longer study times.

## Figures and Tables

**Table 1:** Summary of the studies on the effectiveness of COVID-19 vaccines.

**The Effectiveness of COVID-19 Vaccines among General Population**										
**First Author/Country**	**Study Design**	**No. of Vaccinated/No. of Unvaccinated**	**Age (Years)**	**Vaccine**	**Outcomes**	**Days after the 1st Dose**	**VE of 1st Dose** **(95% CI)**	**Days after the 2nd Dose**	**VE of 2nd Dose** **(95% CI)**	**Variants Involved**
Dagan et al./Israel [28]	Cohort study	596,618/596,618	≥16	BNT162b2	Overall infection	14-20	46% (40–51%)	≥7	92% (88–95%)	B.1.1.7
		596,618/596,618	≥16	BNT162b2	Symptomatic infection	14–20	57% (50–63%)	≥7	94% (87–98%)	B.1.1.7
		596,618/596,618	≥16	BNT162b2	Hospitalization	14–20	74% (56–86%)	≥7	87% (55–100%)	B.1.1.7
		596,618/596,618	≥16	BNT162b2	Critical disease	14–20	62% (39–80%)	≥7	92% (75–100%)	B.1.1.7
		596,618/596,618	≥16	BNT162b2	Death	14–20	72% (19–100%)	N/A	N/A	B.1.1.7
Haas et al./Israel [33]	Cohort study	4,714,932/1,823,979 ^a^	≥16	BNT162b2	Overall infection	N/A	N/A	≥7	95.3% (94.9–95.7%)	B.1.1.7
		4,714,932/1,823,979 ^a^	≥16	BNT162b2	Asymptomatic infection	N/A	N/A	≥7	91.5% (90.7–92.2%)	B.1.1.7
		4,714,932/1,823,979 ^a^	≥16	BNT162b2	Symptomatic infection	N/A	N/A	≥7	97.0% (96.7–97.2%)	B.1.1.7
		4,714,932/1,823,979 ^a^	≥16	BNT162b2	Hospitalization	N/A	N/A	≥7	97.2% (96.8–97.5%)	B.1.1.7
		4,714,932/1,823,979 ^a^	≥16	BNT162b2	Critical disease	N/A	N/A	≥7	97.5% (97.1–97.8%)	B.1.1.7
		4,714,932/1,823,979 ^a^	≥16	BNT162b2	Death	N/A	N/A	≥7	96.7% (96.0–97.3%)	B.1.1.7
Pritchard et al./UK [42]	Case-control study	67,738/192,224	≥16	BNT162b2	Overall infection	≥21	66% (60–71%)	≥1	80% (73–85%)	B.1.1.7
		123,850/192,224	≥16	ChAdOx1	Overall infection	≥21	61% (54–68%)	≥1	79% (65–88%)	B.1.1.7
		67,738/192,224	≥16	BNT162b2	Asymptomatic infection	≥21	47% (35–57%)	≥1	58% (43–69%)	B.1.1.7
		123,850/192,224	≥16	ChAdOx1	Asymptomatic infection	≥21	47% (33–58%)	≥1	61% (27–79%)	B.1.1.7
		67,738/192,224	≥16	BNT162b2	Symptomatic infection	≥21	78% (72–83%)	≥1	95% (91–98%)	B.1.1.7
		123,850/192,224	≥16	ChAdOx1	Symptomatic infection	≥21	71% (62–78%)	≥1	92% (78–97%)	B.1.1.7
Pawlowski et al./US [40]	Cohort study	51,795/51,795	≥18	BNT162b2	Overall infection	≥14	61.0% (50.8–69.2%)	≥7	86.1% (82.4–89.1%)	
		16,471/16,471	≥18	mRNA-1273	Overall infection	≥14	66.6% (51.9–77.3%)	≥7	93.3% (85.7–97.4%)	
		51,795/51,795	≥18	BNT162b2	Hospitalization	N/A	N/A	≥7	88.8% (75.5–95.7%)	
		16,471/16,471	≥18	mRNA-1273	Hospitalization	N/A	N/A	≥7	86.0% (71.6–93.9%)	
		51,795/51,795	≥18	BNT162b2	ICU admission	N/A	N/A	≥7	100.0% (51.4–100%)	
		16,471/16,471	≥18	mRNA-1273	ICU admission	N/A	N/A	≥7	100.0% (43.3–100%)	
Björk et al./Sweden [23] ^b^	Cohort study	26,587/779,154	18–64	BNT162b2	Overall infection	≥14	42% (14–63%)	≥7	86% (72–94%)	
Abu-Raddad et al./Qatar [9]	Case-control study	51,324/162,434	N/A	BNT162b2	Infection of B.1.1.7	≥1	29.5% (22.9–35.5%)	≥14	89.5% (85.9–92.3%)	B.1.1.7
		51,324/162,434	N/A	BNT162b2	Infection of B.1.351	≥1	16.9% (10.4–23.0%)	≥14	75.0% (70.5–78.9%)	B.1.351
		51,324/162,434	N/A	BNT162b2	Severe, critical, or fatal disease caused by the B.1.1.7 variant	≥1	54.1% (26.1–71.9%)	≥14	100.0% (81.7–100.0%)	B.1.1.7
		51,324/162,434	N/A	BNT162b2	Severe, critical, or fatal disease caused by the B.1.351 variant	≥1	0.0% (0.0–19.0%)	≥14	100.0% (73.7–100.0%)	B.1.351
		51,324/162,434	N/A	BNT162b2	Severe, critical, or fatal disease caused by any SARS–CoV–2	≥1	39.4% (24.0–51.8%)	≥14	97.4% (92.2–99.5%)	
Lopez Bernal et al./UK [46]	Case-control study	5553/24,706	≥80	BNT162b2	Symptomatic infection	28–34	70% (59–78%)	≥14	89% (85–93%)	B.1.1.7
		12,122/51,955	≥70	BNT162b2	Symptomatic infection	28–34	61% (51–69%)	N/A	N/A	B.1.1.7
		10,544/51,955	≥70	ChAdOx1	Symptomatic infection	28–34	60% (41–73%)	N/A	N/A	B.1.1.7
		3484/8892	≥80	BNT162b2	Hospitalization	≥14	Further 43% (33–52%) ^c^	N/A	N/A	B.1.1.7
		688/8892	≥80	ChAdOx1	Hospitalization	≥14	Further 37% (3–59%) ^c^	N/A	N/A	B.1.1.7
		1846/8096	≥80	BNT162b2	Death	≥14	Further 51% (37–62%) ^c^	N/A	N/A	B.1.1.7
Vasileiou et al./UK [50]	Cohort study	1,331,993/3,077,595	≥18	BNT162b2, ChAdOx1	Hospitalization	28–34	89% (83–92%)	N/A	N/A	
		711,839/3,077,595	≥18	BNT162b2	Hospitalization	28–34	91% (85–94%)	N/A	N/A	
		620,154/3,077,595	≥18	ChAdOx1	Hospitalization	28–34	88% (75–94%)	N/A	N/A	
Glampson et al./UK [32] ^b^	Cohort study	223,201/1,797,286	≥16	BNT162b2	Overall infection	28	78% (73–82%)	N/A	N/A	
		163,452/1,797,286	≥16	ChAdOx1	Overall infection	28	74% (65–81%)	N/A	N/A	
Corchado–Garcia et al./US [27] ^b^	Cohort study	2195/21,950	≥18	Ad26.COV2.S	Overall infection	≥14	76.7% (30.3–95.3%)	N/A	N/A	
Lopez Bernal et al./UK [21] ^b^	Cohort study	6108/38,038	≥70	BNT162b2	Death	≥21	Further 44% (32–53%) ^c^	≥7	Further 69% (31–86%)	
		3950/38,038	≥70	ChAdOx1	Death	≥21	Further 55% (41–66%) ^c^	N/A	N/A	
Chung et al./Canada [26] ^b^	Case-control study	21,272/302,761	≥16	BNT162b2, mRNA-1273	Symptomatic infection	≥14	60% (57–64%)	≥7	91% (89–93%)	
		18,332/302,761	≥16	BNT162b2	Symptomatic infection	≥14	59% (55–62%)	≥7	91% (88–93%)	
		2940/302,761	≥16	mRNA-1273	Symptomatic infection	≥14	72% (63–80%)	≥7	94% (86–97%)	
		21,272/302,761	≥16	BNT162b2, mRNA-1273	Hospitalization, or death	≥14	70% (60–77%)	≥7	98% (88–100%)	
		18,332/302,761	≥16	BNT162b2	Hospitalization, or death	≥14	69% (59–77%)	≥0	96% (82–99%)	
		2940/302,761	≥16	mRNA-1273	Hospitalization, or death	≥14	73% (42–87%)	≥0	96% (74–100%)	
		21,272/302,761	≥16	BNT162b2, mRNA-1273	Symptomatic infection of B.1.1.7	≥14	61% (56–66%)	≥7	90% (85–94%)	B.1.1.7
		21,272/302,761	≥16	BNT162b2, mRNA-1273	Symptomatic infection of B.1.351 or P.1	≥14	43% (22–59%)	≥7	88% (61–96%)	B.1.351, P.1
		21,272/302,761	≥16	BNT162b2, mRNA-1273	Hospitalization, or death of B.1.1.7	≥14	59% (39–73%)	≥0	94% (59–99%)	B.1.1.7
		21,272/302,761	≥16	BNT162b2, mRNA-1273	Hospitalization, or death of B.1.351 or P.1	≥14	56% (−9–82%)	≥0	100%	B.1.351, P.1
Skowronski et al./Canada [46] ^b^	Case-control study	12,471/4522	≥70	BNT162b2, mRNA-1273	Overall infection	≥21	65% (58–71%)	N/A	N/A	
		10,569/4522	≥70	BNT162b2	Overall infection	≥21	64% (57–71%)	N/A	N/A	
		1882/4522	≥70	mRNA-1273	Overall infection	≥21	71% (56–81%)	N/A	N/A	
		12,471/4522	≥70	BNT162b2, mRNA-1273	Infection of non-variant of concern	≥21	72% (58–81%)	N/A	N/A	Non-variant of concern
		12,471/4522	≥70	BNT162b2, mRNA-1273	Infection of B.1.1.7	≥21	67% (57–75%)	N/A	N/A	B.1.1.7
		12,471/4522	≥70	BNT162b2, mRNA-1273	Infection of P.1	≥21	61% (45–72%)	N/A	N/A	P.1
Emborg et al./Denmark [11] ^b^	Cohort study	473,957/390,139 ^d^		BNT162b2	Overall infection	N/A	N/A	7	82% (79–84%)	
		79,185/19,348	≥85	BNT162b2	Overall infection	N/A	N/A	7	77% (50–89%)	
		473,957/390,139 ^d^		BNT162b2	Hospitalization	N/A	N/A	7	93% (89–96%)	
		473,957/390,139 ^d^		BNT162b2	Death	N/A	N/A	7	94% (90–96%)	
Ranzani et al./Brazil [43] ^b^	Case-control study	4854/11,046	≥70	CoronaVac	Infection	N/A	N/A	≥14	41.6% (26.9–53.3%)	P.1
Lopez Bernal et al./UK [20] ^b^	Case-control study	79,665/107,727	≥16	BNT162b2, ChAdOx1	Symptomatic infection of B.1.1.7	≥21	48.7% (45.5–51.7%)	≥14	87.5% (85.1–89.5%)	B.1.1.7
		25,148/107,727	≥16	BNT162b2	Symptomatic infection of B.1.1.7	≥21	47.5% (41.6–52.8%)	≥14	93.7% (91.6–95.3%)	B.1.1.7
		54,517/107,727	≥16	ChAdOx1	Symptomatic infection of B.1.1.7	≥21	48.7% (45.2–51.9%)	≥14	74.5% (68.4–79.4%)	B.1.1.7
		79,665/107,727	≥16	BNT162b2, ChAdOx1	Symptomatic infection of B.1.617.2	≥21	30.7% (25.2–35.7%)	≥14	79.6% (76.7–82.1%)	B.1.617.2
		25,148/107,727	≥16	BNT162b2	Symptomatic infection of B.1.617.2	≥21	35.6% (22.7–46.4%)	≥14	88.0% (85.3–90.1%)	B.1.617.2
		54,517/107,727	≥16	ChAdOx1	Symptomatic infection of B.1.617.2	≥21	30.0% (24.3–35.3%)	≥14	67.0% (61.3–71.8%)	B.1.617.2
Vahidy et al./US [49] ^b^	Cohort study	27,203/63,931	All	BNT162b2, mRNA-1273	Hospitalization	>14	77% (71–82%)	>7	96% (95–99%)	B.1, B.1.2, B.1.596, B.1.1.7
		27,203/63,931	All	BNT162b2, mRNA-1273	Death	>14	64.2% (13.0–85.2%)	>7	98.7% (91.0–99.8%)	B.1, B.1.2, B.1.596, B.1.1.7
Baum et al./Finland [18] ^b^	Cohort study	758,437/95,719	≥70	BNT162b2, mRNA-1273	Overall infection	21–27	41% (25–54%)	≥7	75% (65–82%)	B.1.1.7
	Cohort study	758,437/95,719	≥70	BNT162b2, mRNA-1273	Hospitalization	21–27	57% (24–75%)	≥7	93% (70–98%)	B.1.1.7
Chodick et al./Israel [24]	Cohort study	503,875 (351,897 had follow-up data for days 13 to 24)	≥16	BNT162b2	Overall infection	13–24	51.4% (16.3–71.8%)	N/A	N/A	
Chodick et al./Israel [25]	Cohort study	1,178,597 (872,454 reach protection period)	≥16	BNT162b2	Overall infection	N/A	N/A	7–27	90% (79–95%)	
		1,178,597 (872,454 reach protection period)	≥16	BNT162b2	Symptomatic infection	N/A	N/A	7–27	94% (88–97%)	
Flacco et al./Italy [31]	Cohort study	69,539/175,687	≥18	BNT162b2, ChAdOx1, mRNA-1273	Overall infection		84% (80–87%)	≥14	98% (97–99%)	B.1.1.7
		47,654/175,687	≥18	BNT162b2	Overall infection	≥14	55% (40–66%)	≥14	98% (96–99%)	B.1.1.7
		16,997/175,687	≥18	ChAdOx1	Overall infection	≥21	95% (92–97%)	≥14	N/A	B.1.1.7
		4888/175,687	≥18	mRNA-1273	Overall infection	≥14	93% (74–98%)	≥14	100%	B.1.1.7
		69,539/175,687	≥18	BNT162b2, ChAdOx1, mRNA-1273	Hospitalization		69% (51–81%)	≥14	99% (96–100%)	B.1.1.7
		47,654/175,687	≥18	BNT162b2	Hospitalization	≥14	N/A	≥14	99% (96–100%)	B.1.1.7
		16,997/175,687	≥18	ChAdOx1	Hospitalization	≥21	100%	≥14	N/A	B.1.1.7
		4888/175,687	≥18	mRNA-1273	Hospitalization	≥14	N/A	≥14	100%	B.1.1.7
		69,539/175,687	≥18	BNT162b2, ChAdOx1, mRNA-1273	Death		73% (−10–93%)	≥14	98% (88–100%)	B.1.1.7
		47,654/175,687	≥18	BNT162b2	Death	≥14	N/A	≥14	98% (87–100%)	B.1.1.7
		16,997/175,687	≥18	ChAdOx1	Death	≥21	100%	≥14	N/A	B.1.1.7
		4888/175,687	≥18	mRNA-1273	Death	≥14	N/A	≥14	100%	B.1.1.7
**The Effectiveness of COVID-19 Vaccines among Healthcare Workers**										
**First Author/Country**	**Study Design**	**No. of Vaccinated/No. of Unvaccinated**		**Vaccine**	**Outcomes**	**Days after the 1st Dose**	**VE of 1st Dose** **(95% CI)**	**Days after the 2nd Dose**	**VE of 2nd Dose** **(95% CI)**	**Variants Involved**
Jones et al./UK [36]	Cohort study	5524/3252		BNT162b2	Asymptomatic infection	≥12	75%	N/A	N/A	B.1.1.7
Fabiani et al./Italy [30]	Cohort study	5333/1090		BNT162b2	Overall infection	14–21	84.1% (39.7–95.8%)	≥7	95.1% (62.4–99.4%)	
		5333/1090		BNT162b2	Symptomatic infection	14–21	83.3% (14.8–96.7%)	≥7	93.7% (50.8–99.2%)	
Hall et al./UK [34]	Cohort study	20,641/2683		BNT162b2	Overall infection	21	72% (58–86%)	≥7	86% (76–97%)	B.1.1.7
Pilishvii et al./US [41]	Case–control study	1201/642		BNT162b2, mRNA-1273	Symptomatic infection	≥14	81.7% (74.3–86.9%)	≥7	93.5% (86.5–96.9%)	
Swift et al./US [47]	Cohort study	44,498/21,932		BNT162b2	Overall infection	>14	78.1% (71.1–82.0%)	>14	96.8% (95.3–97.8%)	
		4722/21,932		mRNA-1273	Overall infection	>14	91.2% (80.6–96.1%)	>14	98.6% (90.1–99.8%)	
Bianchi et al./Italy [22]	Cohort study	1607/427		BNT162b2	Overall infection	14–20	61.9% (19.2–82.0%)	≥7	96.0% (82.2–99.1%)	
Daniel et al./UK [29]	Cohort study	14,265/8969		BNT162b2, mRNA-1273	Overall infection	≥1	30%	≥7/≥14 ^e^	97%	
Benenson et al./Israel [19]	Cohort study	5297/955		BNT162b2	Overall infection	14–20	40%	7–13	94%	B.1.1.7
Amit et al./Israel [15]	Cohort study	7214/1895		BNT162b2	Overall infection	15–28	75% (72–84%)	N/A	N/A	
		7214/1895		BNT162b2	Symptomatic infection	15–28	85% (71–92%)	N/A	N/A	
Lumley et al./UK [38] ^b^	Cohort study	11,023/2086		BNT162b2, ChAdOx1	Overall infection	N/A	64% (50–74%)	N/A	90% (62–98%)	B.1.1.7
		11,023/2086		BNT162b2, ChAdOx1	Symptomatic infection	N/A	67% (48–79%)	N/A	100%	B.1.1.7
Angel et al./Israel [16]	Cohort study	5953/757		BNT162b2	Asymptomatic infection	7–21	36% (−51–69%)	>7	86% (69–93%)	
		5953/757		BNT162b2	Symptomatic infection	7–21	89% (83–94%)	>7	97% (94–99%)	
Moustsen-Helms et al./Denmark [39] ^a^	Cohort study	91,865/239,174		BNT162b2	Overall infection	>14	17% (4–28%)	>7	90% (82–95%)	
Emborg et al./Denmark [11] ^b^	Cohort study	119,951/305,848		BNT162b2	Overall infection	N/A	N/A	7	80% (77–83%)	
Azamgarhi et al./UK [17]	Cohort study	1409/851		BNT162b2	Overall infection	≥14	70% (6–91%)	N/A	N/A	
Thompson et al./US [48]	Cohort study	3179/796		BNT162b2, mRNA-1273	Overall infection	≥14	81% (64–90%)	≥14	91% (76–97%)	B.1.429, B.1.427, B.1.1.7, P.2
				BNT162b2	Overall infection	≥14	80% (60–90%)	≥14	93% (78–98%)	B.1.429, B.1.427, B.1.1.7, P.2
				mRNA-1273	Overall infection	≥14	83% (40–95%)	≥14	82% (20–96%)	B.1.429, B.1.427, B.1.1.7, P.2
Hitchings et al./Brazil [35] ^b^	Case–control study	47,170/5983		CoronaVac	Symptomatic infection	N/A	N/A	≥14	36.8% (54.9−74.2%)	P.1
Shrestha et al./US [44] ^b^	Cohort study	28,223/18,643		BNT162b2, mRNA-1273	Overall infection	14	95.0% (93.0–96.4%)	≥14	97.1% (94.3–98.5%)	
**The Effectiveness of COVID-19 Vaccines among Residents of a Long-Term Care Facility, Subjects with Comorbidity, Subjects with Chronic Illness, or Elderly People (≥65 Years) Requiring Personal Care**										
**First Author/Country**	**Study Design**	**No. of Vaccinated/No. of Unvaccinated**	**Participants**	**Vaccine**	**Outcomes**	**Days after the 1st Dose**	**VE of 1st Dose** **(95% CI)**	**Days after the 2nd Dose**	**VE of 2nd Dose** **(95% CI)**	**Variants Involved**
Moustsen-Helms et al./Denmark [39] ^a^	Cohort study	37,172/1868	RLCF	BNT162b2	Overall infection	>14	21% (−11–44%)	>7	64% (14–84%)	
Shrotri et al./UK [45]	Cohort study	9160/1252	RLCF	BNT162b2, ChAdOx1	Overall infection	35–48	62% (23–81%)	N/A	N/A	
		3022/1252	RLCF	BNT162b2	Overall infection	35–48	65% (29–83%)	N/A	N/A	
		6138/1252	RLCF	ChAdOx1	Overall infection	35–48	68% (34–85%)	N/A	N/A	
Emborg et al./Denmark [11] ^b^	Cohort study	42744/3357	RLCF	BNT162b2	Overall infection	N/A	N/A	7	53% (29–69%)	
		51,311/10,494	65PHC	BNT162b2	Overall infection	N/A	N/A	7	86% (78–91%)	
		180,766/51,092	SCC	BNT162b2	Overall infection	N/A	N/A	7	71% (58–80%)	
		42,744/3357	RLCF	BNT162b2	Hospitalization	N/A	N/A	7	75% (49–89%)	
		51,311/10,494	65PHC	BNT162b2	Hospitalization	N/A	N/A	7	87% (70–95%)	
		180,766/51,092	SCC	BNT162b2	Hospitalization	N/A	N/A	7	81% (49–93%)	
		42,744/3357	RLCF	BNT162b2	Death	N/A	N/A	7	89% (81–93%)	
		51,311/10,494	65PHC	BNT162b2	Death	N/A	N/A	7	97% (88–99%)	
Mazagatos et al./Spain [12]	Case-control study	300,133/38,012	RLCF (≥65 y/o)	BNT162b2, mRNA-1273	Overall infection	>14	50.5% (37.1–61.1%)	≥7/≥14 ^e^	71.4% (55.7–81.5%)	
		300,133/38,012	RLCF (≥65 y/o)	BNT162b2, mRNA-1273	Asymptomatic infection	>14	58.0% (41.7–69.7%)	≥7/≥14 ^e^	69.7% (47.7–82.5%)	
		300,133/38,012	RLCF (≥65 y/o)	BNT162b2, mRNA-1273	Hospitalization	>14	53.0% (25.7–70.3%)	≥7/≥14 ^e^	88.4% (74.9–94.7%)	
		300,133/38,012	RLCF (≥65 y/o)	BNT162b2, mRNA-1273	Death	>14	55.6% (26.6–73.2%)	≥7/≥14 ^e^	97.0% (91.7–98.9%)	

N/A: not available, RLCF: residents of long-term care facilities, SCC: subjects with comorbidity or chronic illness, 65PHC: individuals 65 years and older living at home but requiring practical help and personal care, y/o: years old. ^a^ No. of fully vaccinated /No. of not fully vaccinated. ^b^ Preprint. ^c^ On top of the protection against symptomatic disease. ^d^ All priority groups for vaccines (individual ≥85 years of age, healthcare workers, residents of long-term care facilities, subjects with comorbidity or chronic illness, individuals ≥65 years living at home but requiring practical help and personal care). ^e^ ≥7 days after the second dose for BNT162b2 and ≥14 days for mRNA-1273.

## Supplementary Materials

The following are available online at https://www.mdpi.com/article/10.3390/vaccines9121489/s1, Figure S1: Vaccine effectiveness against critical disease, Table S1: Characteristics of the included studies, Table S2: Risk of bias assessment by ROBINS-I, Table S3: Summary of the studies on the effectiveness of COVID-19 vaccines across age groups.
