# Supplementary material for: Effectiveness of the WHO-Authorized COVID-19 Vaccines: A Rapid Review of Global Reports till 30 June 2021
Source: Vaccines (Basel). 2021 Dec 16;9(12):1489. doi: 10.3390/vaccines9121489 (PMC8708265; doi:10.3390/vaccines9121489)
Supplement: Supplementary file 1 [file vaccines-09-01489-s001.zip › vaccines-1485258-supplementary.pdf]

Supplementary Figure

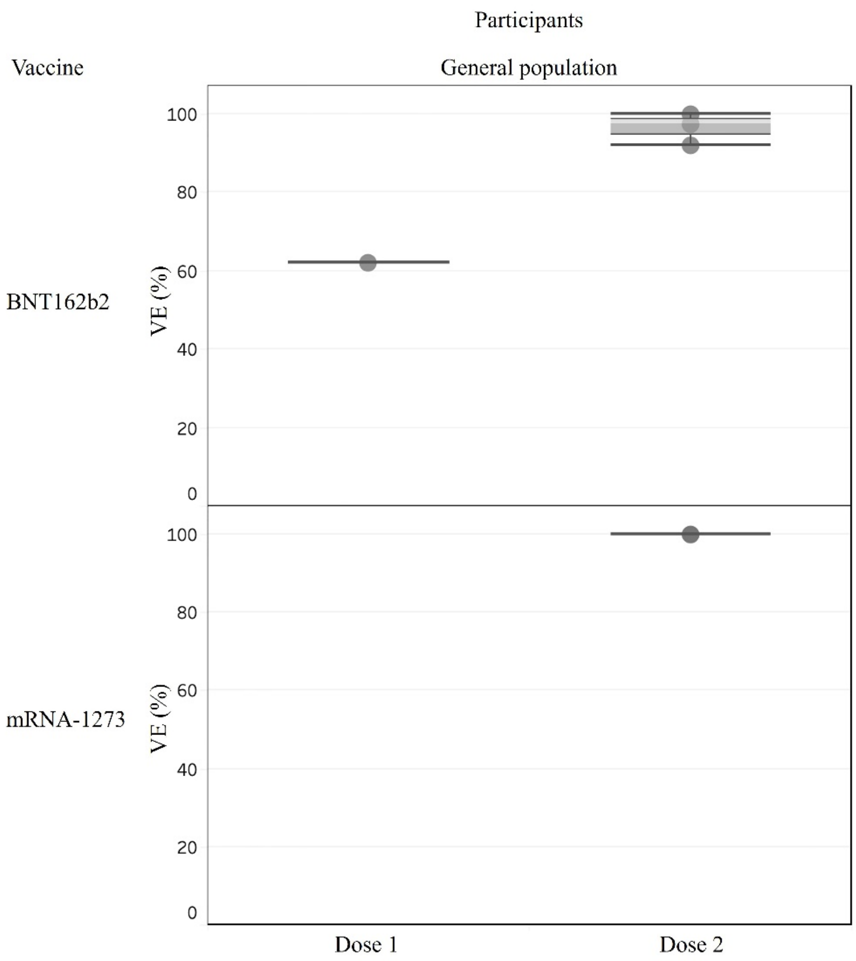

Figure S1. Vaccine effectiveness against critical disease

Supplementary Tables

Table S1. Characteristics of the included studies (n=39)

Country of origin	
The United Kingdom	12
The United States	7
Israel	7
Italy	3
Denmark	2
Canada	2
Brazil	2
Qatar	1
Sweden	1
Spain	1
Finland	1
Study design	
Cohort	30
Case control	9
Vaccine	
BNT162b2	36
mRNA-1273	12
ChAdOx1	10
CoronaVac	2
Ad26.COV2.S	1
Study participants	
General population	22
Healthcare workers	17
Residents of long-term care facility	4
Individuals with comorbidities or chronic illnesses	2
Outcomes of interest	
Infection	29
Asymptomatic infection	5
Symptomatic infection	13
Hospitalization	11
Severe illness	4
Death	9
SARS-CoV-2 variants of concern included	
B1.1.7	16
B.1.351	2
P.1	4
B.1.617.2	1

Table S2. Risk of bias assessment by ROBINS-I

	Bias due to confounding	Bias in selection of participants into the study	Bias in classification of interventions	Bias due to deviations from intended interventions	Bias due to missing data	Bias in measurement of outcomes	Bias in selection of the reported result	Overall bias
Dagan et al.[27]	Moderate	Low	Low	Low	Low	Low	Low	Moderate
Haas et al.[32]	Moderate	Low	Low	Low	Low	Low	Low	Moderate
Pritchard et al.[41]	Moderate	Low	Low	Low	Low	Low	Low	Moderate
Pawlowski et al.[39]	Moderate	Low	Low	Low	Low	Low	Low	Moderate
Björk et al.[22]	Serious	Low	Low	Low	Moderate	Low	Low	Serious
Abu-Raddad et al.[8]	Moderate	Low	Low	Low	Low	Low	Low	Moderate
Lopez Bernal et al.[61]	Moderate	Low	Low	Low	Low	Low	Low	Moderate
Vasileiou et al.[49]	Moderate	Low	Low	Low	Low	Low	Low	Moderate
Glampson et al.[31, 51]	Moderate	Low	Low	Low	Low	Low	Low	Moderate
Corchado-Garcia et al. [26]	Moderate	Low	Low	Low	Low	Low	Low	Moderate
Lopez Bernal et al.[20]	Moderate	Low	Low	Low	Low	Low	Low	Moderate
Chung et al.[25]	Moderate	Low	Low	Low	Low	Low	Low	Moderate
Skowronski et al.[45, 52]	Moderate	Low	Low	Low	Moderate	Low	Low	Moderate
Jones et al.[35]	Moderate	Low	Low	Low	Low	Low	Low	Moderate
Fabiani et al.[29]	Moderate	Low	Low	Low	Low	Low	Low	Moderate
Hall et al.[33]	Moderate	Low	Low	Low	Low	Low	Low	Moderate
Pilishvii et al.[40]	Moderate	Low	Low	Low	Low	Low	Low	Moderate

Swift et al.[46]	Moderate	Low	Low	Low	Low	Low	Low	Moderate
Bianchi et al.[21]	Moderate	Low	Low	Low	Low	Low	Low	Moderate
Daniel et al.[28]	Serious	Serious	Low	Low	Low	Low	Low	Serious
Benenson et al.[18]	Critical	Critical	Low	Low	Low	Low	Low	Critical
Amit et al.[14]	Moderate	Low	Low	Low	Low	Low	Low	Moderate
Lumley et al.[37, 60]	Moderate	Low	Low	Low	Low	Low	Low	Moderate
Angel et al.[15]	Moderate	Low	Low	Low	Low	Low	Low	Moderate
Moustsen-Helms et al.[38]	Moderate	Low	Low	Low	Low	Low	Low	Moderate
Shrotri et al.[50]	Moderate	Low	Low	Low	Low	Low	Low	Moderate
Emborg et al.[10]	Moderate	Low	Low	Low	Low	Low	Low	Moderate
Ranzani et al.[42]	Moderate	Low	Low	Low	Low	Low	Low	Moderate
Mazagatos et al.[11]	Moderate	Low	Low	Low	Low	Low	Low	Moderate
Azamgarhi et al.[16]	Moderate	Low	Low	Low	Low	Low	Low	Moderate
Thompson et al.[47]	Moderate	Low	Low	Low	Moderate	Low	Low	Moderate
Lopez Bernal et al.[19]	Moderate	Low	Low	Low	Low	Low	Low	Moderate
Hitchings et al.[34]	Moderate	Low	Low	Low	Low	Low	Low	Moderate
Shrestha et al.[43]	Moderate	Low	Low	Low	Low	Low	Low	Moderate
Vahidy et al.[48]	Moderate	Low	Low	Low	Low	Low	Low	Moderate
Baum et al.[17]	Moderate	Low	Low	Low	Low	Low	Low	Moderate
Chodick et al.[23]	Moderate	Low	Low	Low	Low	Low	Low	Moderate
Chodick et al.[24]	Moderate	Low	Low	Low	Low	Low	Low	Moderate
Flacco et al.[30]	Moderate	Low	Low	Low	Low	Low	Low	Moderate

Table S3. Summary of the studies on the effectiveness of COVID-19 vaccines across age groups

Study first author / Country	Study design	No. of vaccinated / No. of unvaccinated	Participants	Age	Vaccine	Outcome	Days after the 1st dose	VE of 1st dose (95% CI)	Days after the 2nd dose	VE of 2nd dose (95% CI)	Variants involved
Dagan et al./ Israel[27]	Cohort study	596618/ 596618	GP	≥16	BNT162b2	Overall infection	14–20	46% (40%–51%)	≥7	92% (88%–95%)	B.1.1.7
		213090/ 213090	GP	16–39	BNT162b2	Overall infection	14–20	49% (41%–57%)	≥7	94% (87%–93%)	B.1.1.7
		304514/ 304514	GP	40–69	BNT162b2	Overall infection	14–20	47% (40%–55%)	≥7	90% (82%–95%)	B.1.1.7
		79014/ 79014	GP	≥70	BNT162b2	Overall infection	14–20	22% (-9%–44%)	≥7	95% (87%–100%)	B.1.1.7
		596618/ 596618	GP	≥16	BNT162b2	Symptomatic infection	14–20	57% (50%–63%)	≥7	94% (87%–98%)	B.1.1.7
		213090/ 213090	GP	16–39	BNT162b2	Symptomatic infection	14–20	57% (46%–68%)	≥7	99% (96%–100%)	B.1.1.7
		304514/ 304514	GP	40–69	BNT162b2	Symptomatic infection	14–20	59% (50%–67%)	≥7	90% (75%–98%)	B.1.1.7
		79014/ 79014	GP	≥70	BNT162b2	Symptomatic infection	14–20	44% (19%–64%)	≥7	98% (90%–100%)	B.1.1.7
Haas et al./ Israel[32]	Cohort study	4714932/ 1823979 [a]	GP	≥16	BNT162b2	Overall infection	N/A	N/A	≥7	95.3% (94.9%–95.7%)	B.1.1.7
		2290820/ 1356028 [a]	GP	16–44	BNT162b2	Overall infection	N/A	N/A	≥7	96.1% (95.7%–96.5%)	B.1.1.7
		1408492/ 355606 [a]	GP	45–64	BNT162b2	Overall infection	N/A	N/A	≥7	94.9% (94.2%–95.5%)	B.1.1.7
		1015620/ 112345 [a]	GP	≥65	BNT162b2	Overall infection	N/A	N/A	≥7	94.8% (93.9%–95.5%)	B.1.1.7
		4714932/ 1823979 [a]	GP	≥16	BNT162b2	Asymptomatic infection	N/A	N/A	≥7	91.5% (90.7%–92.2%)	B.1.1.7
		2290820/ 1356028 [a]	GP	16–44	BNT162b2	Asymptomatic infection	N/A	N/A	≥7	93.6% (92.8%–94.4%)	B.1.1.7

1408492/ 355606 [a]	GP	45–64	BNT162b2	Asymptomatic infection	N/A	N/A	≥7	90.8% (89.6%–91.9%)	B.1.1.7
1015620/ 112345 [a]	GP	≥65	BNT162b2	Asymptomatic infection	N/A	N/A	≥7	88.5% (86.4%–90.3%)	B.1.1.7
4714932/ 1823979 [a]	GP	≥16	BNT162b2	Symptomatic infection	N/A	N/A	≥7	97.0% (96.7%–97.2%)	B.1.1.7
2290820/ 1356028 [a]	GP	16–44	BNT162b2	Symptomatic infection	N/A	N/A	≥7	97.6% (97.3%–97.8%)	B.1.1.7
1408492/ 355606 [a]	GP	45–64	BNT162b2	Symptomatic infection	N/A	N/A	≥7	96.7% (96.3%–97.0%)	B.1.1.7
1015620/ 112345 [a]	GP	≥65	BNT162b2	Symptomatic infection	N/A	N/A	≥7	96.4% (95.9%–97.0%)	B.1.1.7
4714932/ 1823979 [a]	GP	≥16	BNT162b2	Hospitalization	N/A	N/A	≥7	97.2% (96.8%–97.5%)	B.1.1.7
2290820/ 1356028 [a]	GP	16–44	BNT162b2	Hospitalization	N/A	N/A	≥7	98.1% (97.3%–98.7%)	B.1.1.7
1408492/ 355606 [a]	GP	45–64	BNT162b2	Hospitalization	N/A	N/A	≥7	97.6% (97.1%–98.1%)	B.1.1.7
1015620/ 112345 [a]	GP	≥65	BNT162b2	Hospitalization	N/A	N/A	≥7	96.8% (96.2%–97.3%)	B.1.1.7
4714932/ 1823979 [a]	GP	≥16	BNT162b2	Critical disease	N/A	N/A	≥7	97.5% (97.1%–97.8%)	B.1.1.7
2290820/ 1356028 [a]	GP	16–44	BNT162b2	Critical disease	N/A	N/A	≥7	98.9% (97.6%–99.5%)	B.1.1.7
1408492/ 355606 [a]	GP	45–64	BNT162b2	Critical disease	N/A	N/A	≥7	98.1% (97.5%–98.5%)	B.1.1.7
1015620/ 112345 [a]	GP	≥65	BNT162b2	Critical disease	N/A	N/A	≥7	97.3% (96.8%–97.8%)	B.1.1.7
4714932/ 1823979 [a]	GP	≥16	BNT162b2	Death	N/A	N/A	≥7	96.7% (96.0%–97.3%)	B.1.1.7
2290820/ 1356028 [a]	GP	16–44	BNT162b2	Death	N/A	N/A	≥7	100%	B.1.1.7
1408492/ 355606 [a]	GP	45–64	BNT162b2	Death	N/A	N/A	≥7	95.8% (92.6%–97.6%)	B.1.1.7
1015620/ 112345 [a]	GP	≥65	BNT162b2	Death	N/A	N/A	≥7	96.9% (96.0%–97.6%)	B.1.1.7

Pritchard et al./ UK[41]	Case–control study	67738/ 192224	GP	≥16	BNT162b2	Overall infection	≥ 21	66% (60%–71%)	≥1	80% (73%–85%)	B.1.1.7
		123850/ 192224	GP	≥16	ChAdOx1	Overall infection	≥ 21	61% (54%–68%)	≥1	79% (65%–88%)	B.1.1.7
			GP	<75	BNT162b2, ChAdOx1	Overall infection	≥ 21	60% (54%–65%)	N/A	N/A	B.1.1.7
			GP	≥75	BNT162b2, ChAdOx1	Overall infection	≥ 21	72% (64%–78%)	N/A	N/A	B.1.1.7
Vasileiou et al./ UK[49]	Cohort study	1331993/ 3077595	GP	≥18	BNT162b2, ChAdOx1	Hospitalization	28–34	89% (83%–92%)	N/A	N/A	
		711839/ 3077595	GP	≥18	BNT162b2	Hospitalization	28–34	91% (85%–94%)	N/A	N/A	
		620154/ 3077595	GP	≥18	ChAdOx1	Hospitalization	28–34	88% (75%–94%)	N/A	N/A	
		470960/ 2913484	GP	18–64	BNT162b2, ChAdOx1	Hospitalization	28–34	92% (82%–97%)	N/A	N/A	
		651924/ 107022	GP	65–79	BNT162b2, ChAdOx1	Hospitalization	28–34	93% (73%–98%)	N/A	N/A	
		209109/ 57089	GP	≥80	BNT162b2, ChAdOx1	Hospitalization	28–34	83% (72%–89%)	N/A	N/A	
		359434/ 2913484	GP	18–64	BNT162b2	Hospitalization	28–34	92% (82%–97%)	N/A	N/A	
		315620/ 107022	GP	65–79	BNT162b2	Hospitalization	28–34	93% (73%–98%)	N/A	N/A	
		36785/ 57089	GP	≥80	BNT162b2	Hospitalization	28–34	88% (76%–94%)	N/A	N/A	
		111526/ 2913484	GP	18–64	ChAdOx1	Hospitalization	28–34	100% (NA–100%)	N/A	N/A	
		336304/ 107022	GP	65–79	ChAdOx1	Hospitalization	28–34	100% (NA–100%)	N/A	N/A	
		172324/ 57089	GP	≥80	ChAdOx1	Hospitalization	28–34	81% (60%–91%)	N/A	N/A	

Ranzani et al./ Brazil[42 b]	Case–control study	4854/ 11046	GP	≥70	CoronaVac	Overall infection	N/A	N/A	≥14	41.6%<br>(26.9%–53.3%)	P.1
			GP	70–74	CoronaVac	Overall infection	N/A	N/A	≥14	61.8%<br>(34.8%–77.7%)	P.1
			GP	75–79	CoronaVac	Overall infection	N/A	N/A	≥14	48.9%<br>(23.3%–66.0%)	P.1
			GP	≥80	CoronaVac	Overall infection	N/A	N/A	≥14	28.0%<br>(0.6%–47.9%)	P.1
Chodick et al./ Israel[23]	Cohort study	503875 (351897 had follow–up data for days 13 to 24)	GP	≥16	BNT162b2	Overall infection	13–24	51.4%<br>(16.3%–71.8%)	N/A	N/A	
			GP	<60	BNT162b2	Overall infection	13–24	50.2%<br>(14.1%–71.2%)	N/A	N/A	
			GP	≥60	BNT162b2	Overall infection	13–24	44.5%<br>(4.2%–67.9%)	N/A	N/A	
Chodick et al./ Israel[24]	Cohort study	1178597 (872454 reach protection period)	GP	≥16	BNT162b2	Overall infection	N/A	N/A	7–27	90%<br>(79%–95%)	
			GP	16–44	BNT162b2	Overall infection	N/A	N/A	7–27	92%<br>(83%–96%)	
			GP	45–64	BNT162b2	Overall infection	N/A	N/A	7–27	90%<br>(80%–95%)	
			GP	65–74	BNT162b2	Overall infection	N/A	N/A	7–27	82%<br>(63%–92%)	
			GP	≥75	BNT162b2	Overall infection	N/A	N/A	7–27	82%<br>(61%–91%)	

GP: general population, N/A: not available

[a] No. of fully vaccinated / No. of not fully vaccinated
[b] Preprint
